# Identification of Performance Improvement Objectives After Management of a Mass Shooting Incident: A Retrospective Study

**DOI:** 10.7759/cureus.47529

**Published:** 2023-10-23

**Authors:** Cynthia Leslie, Kevin DiMagno

**Affiliations:** 1 Division of Trauma and Critical Care, Good Samaritan University Hospital, West Islip, USA; 2 Medical School, New York Institute of Technology College of Osteopathic Medicine, Old Westbury, USA

**Keywords:** process & performance improvement, trauma critical care, healthcare policy, disaster response and preparedness, mass shooting

## Abstract

Mass shootings are firearm incidents involving four or more victims at one or more locations close to one another. Although some American College of Surgeons designated trauma centers have the experience and resources to adequately treat mass shooting victims who arrive simultaneously or in close proximity to each other, many others do not. Therefore, the objective of this retrospective case series was to evaluate the effectiveness of the treatment of five consecutive gunshot wound victims who presented to a Level II trauma center within 36 minutes of each other. Lessons learned from that experience were used to identify the most effective pre-hospital and hospital management interventions. Opportunities for performance improvement were analyzed with respect to the current literature and the American College of Surgeons 2022 consensus recommendations for mass shootings.

## Introduction

Mass shootings are a major health problem plaguing the United States. Mass shootings, as defined by both the Congressional Research Service and the FBI, are firearm incidents involving four or more victims at one or more locations close to one another [1]. Homicide is the leading cause of death for Americans younger than 45 years of age [2]. Although homicides are the leading cause of death, mass shootings represented less than 0.1% of all homicides in the United States during the years 2000 to 2016 [3], America continues to have the highest incidence of mass shootings in the world, accounting for 31% of global mass shooting events [4]. The exponential increase in the number of mass shootings has paradoxically occurred in the setting of an overall decrease in violent crime.

A new mass shooting is estimated to occur in the United States every 64 days and has been driven primarily by easy legal and illegal access to firearms [5]. Reaping et al. demonstrated that laxity in state gun laws increased the rate of mass shooting events as a result of higher gun ownership rates [6]. In the United States, Siegel et al. demonstrated that for every one percentage point increase in gun ownership per state, the homicide rate due to firearms increased by 0.9% [7]. Tiderman et al. demonstrated that states with more restrictive gun laws had lower rates of fatal mass shootings [8]. The public health response to mass shootings requires a multifaceted approach involving multiple levels of legislative and medical coordination. Although many hospitals have been designated by local and state municipalities as trauma centers, only some trauma centers are verified by the American College of Surgeons (ACS) as having the experienced teams and resources to adequately treat mass shooting victims who arrive simultaneously or in close proximity to each other [9]. Additionally, responses can vary due to prehospital triage and treatment and communications with the trauma center team [10]. We present a case highlighting the medical response to a mass shooting event with the identification of quality improvement objectives based on a review of the current literature.

On June 13, 2020, a mass shooting occurred in a Long Island, New York suburb, at approximately 0139. Five critically injured patients with penetrating trauma were transported to a 437-bed teaching hospital. At that time, the hospital was an ACS verified Level II trauma center with a high-volume emergency department (ED) and a catchment area of approximately 400,000 people [11]. One trauma surgeon and one trauma physician assistant were on-call in the hospital when the shootings occurred.

## Materials and methods

Five patients were identified who were transported to an ACS Level II trauma center, Good Samaritan University Hospital, following the mass shooting event that took place on June 13, 2020. After the approval by the Good Samaritan University Hospital Institutional Review Board (approval number: 2022.05.17.17) as an exempt study, a retrospective case series was conducted and the five patients' charts were analyzed and reviewed.

Triage information from Emergency Medical Services (EMS) in the field and transfer times from the scene to the hospital were obtained through EMS patient transport reports (PCRs) for all five patients. The data extracted from EMS PCRs included injuries sustained, treatments performed prior to arrival, and transfer times. Data extracted from the electronic health record of the patients included arrival time, ED procedures and treatments, diagnostic studies performed, diagnoses, and patient ED disposition. Information regarding the ED census and staffing was obtained from the daily ED staffing report.

For the literature review, the databases of PubMed, Scopus, and the ACS were searched. Literature that was published between January 2018 and August 2023 using the terms “mass shooting”, “mass casualties”, and “penetrating traumas” was used. The rationale for this literature review sampling frame was the ACS Committee on Trauma Firearms Strategy Team (FAST). These recommendations were designed to develop an effective strategy to reduce firearm injury, death, and disability in 2018 [12].

## Results

Field triage and transfer times to trauma center

The mass shooting occurred in a suburban community located 10 miles from the hospital. Police received a 911 call at 0139 that originated from a residential building. Multiple casualties were suspected but the exact number of victims was not known. Paramedics were dispatched and began their field triage at 0143. Multiple patients with penetrating trauma were found at the scene. Two of the most critically injured had multiple gunshot wounds (GSWs) to the chest, abdomen, and extremities. Before leaving the scene, paramedics pre-activated the highest level designation of injury severity for two of the most critically injured patients. These two patients were each identified as Code T. The patients’ pre-hospital vital signs, injuries, and pre-hospital interventions are summarized in Table 1.

**Table 1:** EMS field triage and clinical interventions performed prior to arrival at the trauma center. GSW: gunshot wound; bpm: beats per minute; BP: blood pressure; resp. rate: respiratory rate; GCS: Glasgow coma scale; RLE: right lower extremity; O2: oxygen; EKG: electrocardiogram; EMS: emergency medical services; CPR: cardiopulmonary resuscitation; IV: intravenous; NA: not available

Patient No.	Age (years)	EMS departure time	ED Arrival time	Transportation Method	Vital signs in the field	Prehospital cardiac arrest	Location of GSWs	Prehospital interventions
1	28	NA	0135	Private Vehicle	NA	NA	NA	NA
2	30	0150	0155	EMS	118 bpm, 108/86 BP, 22 unassisted resp. rate, GCS of 15	No	GSW to right upper leg and right lower leg with arterial bleeding	RLE tourniquet, EKG, IV insertion, Normal saline, O2
3	30	NA	0200	Private Vehicle	NA	NA	NA	NA
4	33	NA	0205	Private Vehicle	NA	NA	NA	NA
5	23	0207	0212	EMS	0 bpm, 0/0 BP, 10 assisted resp. rate, GCS of 3	Yes	GSW Left chest/flank, medial right thigh, right forearm	CPR, IV insertion, King airway, Lucas device

ED triage

As per hospital policy, one fellowship-trained, board-certified trauma surgeon should be in the hospital, at all times. In addition, there should be a dedicated backup trauma surgeon who must report to the hospital within 30 minutes of being called by the in-house trauma surgeon. When the first shooting victim arrived at 0136, the ED contained 34 patients, which included those in the main ED, fast track area, and three trauma rooms. ED staff consisted of two ED attending physicians and two emergency medicine residents in the adult treatment area, and one pediatric attending physician and one emergency medicine resident in the pediatric ED. The three trauma rooms were occupied by patients undergoing treatment for non-traumatic medical conditions.

The first patient arrived at 0136 after being transported in a private car. He had a single GSW to the left lower abdominal quadrant and was immediately triaged as a Code T. The in-house trauma surgeon arrived within five minutes of being notified and determined that the patient had a protected airway, minimal abdominal pain, and was neurologically intact and hemodynamically stable. Moreover, the trajectory of the bullet and the patient’s lack of abdominal pain and tenderness to palpation did not appear to mandate immediate exploratory laparotomy. Advanced trauma life support (ATLS) protocol was initiated, and the patient was monitored closely.

The first ambulance transport arrived at 0155 as a pre-activated Code T from the field. He was in hemorrhagic shock with multiple GSWs to the abdomen and right lower extremity and was immediately intubated by the emergency medicine attending. The backup trauma surgeon was notified and arrived within 20 minutes of the arrival of this second Code T.

Two more trauma patients arrived in private cars five and 10 minutes after the second Code T. Both patients were hemodynamically stable and neurologically intact on arrival. They were immediately triaged as the third and fourth Code T. The third Code T had a GSW to the left buttock and required intubation due to altered mental status and the need for airway control. The fourth Code T had a GSW to the left thigh. A fifth trauma patient arrived by ambulance at 0212, as a pre-activated Code T, with multiple GSWs to the left chest and right upper extremity. CPR had been started at the scene and was continued on arrival to the hospital. When the fifth Code T arrived, both the in-house and backup trauma surgeons were treating other patients. Therefore, a third tier of support was required. As per our trauma policy, the on-call surgical critical care intensivist, who is a trauma surgeon, was called and arrived within 15 minutes. In the meantime, the emergency medicine attendings and residents were instrumental in assuming the care of this patient until the third trauma surgeon arrived.

Definitive trauma management, complications, and outcomes

The first Code T was initially managed nonoperatively due to the bullet’s equivocal trajectory and lack of an abdominal examination mandating immediate exploratory laparotomy. Although the patient remained hemodynamically stable, he subsequently developed abdominal pain which prompted a CT of the abdomen with IV contrast. The CT demonstrated non-physiological, intra-peritoneal fluid, and the patient was taken to surgery at 0332. He was found to have two through and through small bowel injuries which required a small bowel resection with primary anastomosis.

The second Code T presented in hemorrhagic shock and received one unit of packed red blood cells in the trauma room. He was taken to the operating room 44 minutes after presenting to the ED. At surgery, he was seen to have multiple projectile injuries of the small bowel and mesentery, a transverse colon injury, and an expanding Zone 1 retroperitoneal hematoma. Because of the number and complexity of the abdominal injuries, a fourth trauma surgeon was called and immediately agreed to come in from home, to assist in surgery.

The third Code T arrived with a single GSW to the left buttock and underwent a CT angiogram with run-off that was negative for arterial injury. After receiving routine wound care, he was extubated and discharged home 16 hours later. The fourth Code T arrived with a single GSW to the left thigh and remained hemodynamically stable with palpable pulses and soft compartments. Computed tomography of the femur and pelvis were negative for fracture and retained foreign bodies. After receiving routine wound care, he was discharged home 11 hours later. The fifth Code T arrived with CPR in progress. After intubation in the field, he received two ampules each of epinephrine, calcium chloride, and sodium bicarbonate in the hospital. However, return of spontaneous circulation (ROSC) was not achieved, and the patient was pronounced dead at 0217. Trauma management, complications, and outcomes are summarized in Table 2.

**Table 2:** Definitive trauma management, complications, and outcomes of the five mass shooting victims following arrival to the ED. LLQ: left lower quadrant; GSW: gunshot wound; bpm: beats per minute; BP: blood pressure; resp. rate: respiratory rate; GCS: Glasgow coma scale; RLE: right lower extremity; O2: oxygen; EKG: electrocardiogram; HCT: hematocrit; ATLS: advanced trauma life support; CT: computed tomography; OR: operating room; ROSC: return of spontaneous circulation; SICU: surgical intensive care unit ^*^3M Company, Saint Paul, Minnesota, United States

Patient No.	Location of GSW	ED Vital Signs	Interventions at Trauma Center	Disposition	Surgical Interventions	Complications	Outcome
1	Abdomen LLQ	73 bpm, 16 resp. rate, 126/74 BP, GCS 15, HCT 42, lactate 5.8	ATLS protocol. Abdominal examination and CT scans of cervical spine, chest abdomen and pelvis	Arrived in the OR at 0322	Small bowel resection and primary anastomosis	None	Lived
2	Abdomen LLQ, right lower extremity	120 bpm, 12 resp. rate, 104/88 BP, GCS 13	Emergent intubation and exploratory laparotomy	Arrived in the OR at 0239	Small bowel resection. Repair of mesenteric and transverse colon injuries with management of Zone I expanding retroperitoneal hematoma	Re-exploration for necrotic distal duodenum, gastrojejunostomy. ABthera* vacuum placement and subsequent abdominal closure	Lived
3	Left buttock	110 bpm, 22 resp. rate, 123/75 BP, GCS 15	Intubated. CT scans of chest, abdomen and pelvis	Discharged home eleven hours after arrival	Irrigation of buttock wound	None	Lived
4	Posterior Left Thigh	137 bpm, 22 resp. rate, 135/112 BP, GCS 15	CT scans of chest, abdomen, pelvis and CT angiogram of bilateral lower extremities	Extubated in the SICU. Discharged home sixteen hours after arrival	Irrigation of thigh wound	None	Lived
5	Bilateral anterior thorax, left lateral chest wall, RUE, and right upper thigh	0 bpm, 0 resp. rate, no BP, GCS 3	ACLS protocol, unable to achieve ROSC	N/A	N/A	N/A	Died

## Discussion

The United States has experienced a 40% increase in the number of mass shootings over the last 10 years [13], with 2423 mass shootings occurring between 2015 and 2021 [14]. In 2020, Sanchez et al. found that the rate of gun violence in the United States was nearly 10 times higher than that of other comparably industrialized, high-income countries [15]. Of the 23 wealthiest countries in the world, the United States accounted for 80% of firearm-related deaths, and as noted by multiple authors, the physical devastation of firearm-induced penetrating trauma is directly related to the transfer of higher amounts of kinetic energy to tissues and organs [16].

In 2022, the ACS formulated 19 consensus recommendations for healthcare responses to mass shootings. Goolsby et al. assembled three groups, consisting of EMS clinicians, emergency physicians, and surgeons [13]. Each group suggested ways in which medical and surgical responses to mass shooting events could be improved. Eight of the 19 recommendations were unanimously adopted. These were: (i) Regular interdisciplinary training for mass shooting events that include hospitals, EMS, law enforcement, fire departments, and 911 dispatch, (ii) Prior public education for real-time directions from mobile apps or alerts to transport patients to the appropriate hospital, (iii) A staged triage process at the scene and at hospitals to prioritize patients for surgery, (iv) Effective communication between personnel at the scene and at hospitals, (v) A system to track patients from point of injury throughout their care, (vi) Alternative methods to document and input patient details, (vii) Rapidly established well-communicated family reunification sites, and (viii) Mental health services for all responders that are tailored to their specific needs.

The remaining 11 recommendations were also considered vital and included “Stop the Bleed” education for communities, modern systems to reach and recall staff, surgeon participation in triage and operative planning, hospital strategies to rapidly increase capacity, and staffing plans for all categories of hospital leadership and employees.

Several of the 2022 ACS consensus recommendations were already being utilized by the hospital in the present study at the time of the mass shooting. Among these were rapid and effective communications between pre-hospital personnel and the receiving hospital. In this case, paramedics at the scene identified two patients who required Code T designations, thus enabling an early warning system for receiving hospital clinicians. The accuracy of the pre-hospital reports allowed for rapid in-hospital triage and decreased the time to diagnosis and treatment by eliminating clinical redundancy. As an ACS-verified trauma center, the trauma surgeons regularly participated in hospital triage and determined operative management, as recommended by the consensus statement. The hospital was also able to flex and repurpose space in the ED and throughout the institution due to multiple lines of communication between in-house staff leadership and on-call administrators. The hospital participated in a region-wide system that reviewed coordinated hospital communications and capacity awareness and was also in compliance with both the ACS consensus recommendations and the Hartford Consensus III statement [17] through its “Stop the Bleed” outreach to schools, nursing homes, government agencies, and community centers.

However, several issues arose that were not anticipated. Three patients arrived in private cars, within 30 minutes of each other. Although they were quickly and accurately triaged, the use of aliases was cumbersome and initially led to confusion about their identities. As recommended by the ACS, the use of a point-of-injury quick response (QR) code, to be used throughout the patient’s hospitalization, could potentially streamline and clarify patient identification. In addition, an electronic board dedicated solely to trauma patients, that identifies patient injuries, radiographic results, and patients’ locations in the hospital, would also contribute to more accurate patient management and tracking.

Three surgeons were on site when the fifth Code T arrived, but a fourth trauma surgeon was needed to assist in surgery. Although four trauma surgeons responded to five critically injured patients promptly and effectively, that response was predicated on the serendipitous availability of trauma surgeons who were not on call and not in-house. Since the hospital has on-call general and vascular surgeons, the implementation of a centralized call center, responsible for quickly notifying all available surgeons and tracking their response times, would be a valuable addition to the hospital’s current disaster plan. In an analysis of a 2017 mass shooting event, Kuhls et al. recommended the use of a SMART (Sustained Medical and Readiness Trained) system that directly activated multiple surgeons, non-surgeon physicians, healthcare professionals, and military surgeons as part of their agreement with a nearby Air Force Base [18]. Because this trauma center was not affiliated with a nearby military base with trained surgeons available, we reviewed SMART for other activation plans that would be applicable to our trauma center.

Numerous authors, including Goolsby et al., have stressed the importance of creating formal agreements with local law enforcement and military agencies in order to provide additional personnel and surge capacity in the event of overwhelming numbers of casualties [13]. Central to this consensus recommendation is the need for regular interdisciplinary training for mass shooting incidents that mirrors worst-case scenarios, and includes trauma and non-trauma centers, EMS, law enforcement, fire departments, and 911 dispatch. Mental health service “after action” plans, which designate support programs for all responders, should also be a part of these training exercises.

Finally, while the families of all the trauma victims treated at the hospital were expeditiously notified of their admission and outcomes, it is not difficult to imagine a scenario in which social workers and nurses would be inundated by such requests. As a result, the establishment of in-hospital or out-of-hospital family reunification sites would be a valuable addition to the hospital’s current disaster preparedness plan. 

Several performance improvement recommendations and initiatives were formulated by our institution as a result of the mass shooting in June 2020. The institution’s Trauma Performance, Trauma Systems, and Trauma Operating committees independently reviewed all aspects of the events in July and August of 2020. Each shooting victim’s case was primarily reviewed by either the Trauma program manager or a Trauma performance improvement (PI) nurse. Each case subsequently underwent a secondary review by either a trauma surgeon or a surgical sub-specialist. One complex case was reviewed and presented at the monthly Trauma peer review committee meeting and all aspects of patient triage and surgical management were determined to be consistent with the standard of care.

The surge policy and disaster plan have also been reviewed and modified to address the logistics of admitting large numbers of traumatically injured patients simultaneously. As a result, an incident-specific command center is being formulated to track patient admissions and monitor resource allocations. Additionally, a comprehensive performance improvement plan has been drafted in response to the multiple assessments and reviews that were conducted. This plan includes biannual mass shooting mock codes, and annual training for clinical, administrative staff, and non-trauma team members. Although the trauma center is not affiliated with a nearby military base, the SMART (Sustained Medical and Readiness Trained) guidelines were reviewed for activation plans that would be applicable to our trauma center going forward. As of this writing, no other mass shooting events have been presented to our trauma center. However, the incorporation of the 2022 ACS consensus recommendations on mass shootings has heightened institutional awareness and readiness as a whole.

## Conclusions

Mass shootings are devastating societal events that exact a tragic and staggering personal, social, and economic toll. As the number of these occurrences increases, verified ACS trauma centers must adopt comprehensive, multi-faceted approaches that include prevention, education, and post-traumatic physical and mental rehabilitation for survivors. Although the hospital in the current study had written policies in place for a sudden surge in patients and has held simulations with staff and departments, the mass shooting incident of June 13, 2020, mandated the need for improved preparation of all hospital staff. The 2022 ACS consensus recommendations for healthcare responses to mass shooting victims offer valuable guidelines for optimizing the care of such traumatically injured patients. Early implementation of a disaster plan specifically designed to triage and care for mass shooting casualties is highly recommended. This includes the immediate establishment of an incident-specific command center designed to coordinate mobilization and monitor resource allocation. A detailed surge policy designed to accommodate multiple trauma victims is also recommended, as are regular in-hospital mock drills that simulate real-time mass casualty events. Implementation of a SMART system that directly activates multiple surgeons, non-surgeon physicians, and other healthcare personnel is also recommended because it immediately alerts multiple practitioners to the need for additional clinicians. Finally, alliances with local police and military agencies are also strongly encouraged in order to facilitate crowd control, patient identification, and family reunification.

In the aftermath of a mass casualty shooting, the 2022 ACS consensus recommendations strongly encourage an independent institutional review of all patient interventions and outcomes by the trauma PI and trauma system operating committees. Additionally, a regular review of existing trauma policies should be done to determine their applicability to future mass shootings. Although the optimal care of mass shooting victims continues to require intensive study and analysis, the 2022 ACS consensus recommendations are a valuable addition to the current literature. Lessons learned from our hospital’s mass shooting event in June 2020 present a valuable opportunity to align with current, best clinical practices.
